# Mucocutaneous nasal histoplasmosis in an immunocompetent dog

**DOI:** 10.1186/s12917-021-02896-9

**Published:** 2021-05-13

**Authors:** Romy M. Heilmann, Mary B. Nabity, Laura K. Bryan, Audrey K. Cook, Katherine Scott

**Affiliations:** 1 Department of Small Animal Clinical Sciences, College of Veterinary Medicine and Biomedical Sciences, Texas A&M University, College Station, TX 77843 USA; 2 Department for Small Animals, Veterinary Teaching Hospital, College of Veterinary Medicine, University of Leipzig, An den Tierkliniken 23, DE-04103 Leipzig, SN Germany; 3 Department of Veterinary Pathobiology, College of Veterinary Medicine and Biomedical Sciences, Texas A&M University, TAMU 4474, College Station, TX 77843 USA; 4 VCA Alameda East Veterinary Hospital, 9770 E Alameda Ave, Denver, CO 80247 USA

**Keywords:** Antifungal, *Histoplasma capsulatum*, Mycotic rhinitis, Nasal discharge, Nasal planum

## Abstract

**Background:**

*Histoplasma (H.) capsulatum* is a dimorphic fungus, and infection is typically via inhalation of microconidia. After conversion to the yeast phase within the lung, the organism is subsequently disseminated to other tissues by macrophages. Nasal histoplasmosis appears to be a rare condition in dogs.

**Case presentation:**

We report the clinical case of a 4.5-year-old male neutered Cocker spaniel/Poodle mix, 7.7 kg, body condition score 6/9, that presented with a 3-month history of sneezing and left-sided mucoid nasal discharge. The history also included a mild swelling (transient) of the right carpus with a lameness (grade II-III/IV), coinciding with the onset of sneezing and nasal discharge. The dog lived primarily indoors in the Texas Gulf Coast area. On physical examination, the dog was febrile, and the left nostril was swollen, ulcerative, deformed, and hypopigmented. Mandibular lymph nodes were firm and mildly enlarged bilaterally. Mild lymphopenia, thrombocytopenia, and hyperglobulinemia were noted. Thoracic radiographs were unremarkable. Computed tomography and rhinoscopy revealed swelling of the rostral portion of the left and right nasal passages. Cytology and histology of biopsies of the affected nasal tissue showed pyogranulomatous inflammation and yeast organisms consistent with *H. capsulatum*. Weak antigenuria was detected on the MVista *H. capsulatum* antigen test. Treatment with oral itraconazole led to a resolution of the nasal signs and normalization of the appearance of the nostril over 13 weeks, and neither antigenuria nor antigenemia was detected on several recheck examinations. The dog remained in good general and physical condition and showed no signs of disease recurrence more than 6 years after the last examination.

**Conclusion:**

We report a rare case of nasal mucocutaneous histoplasmosis in an immunocompetent dog, with an excellent clinical response to oral itraconazole. This case documents that histoplasmosis in dogs can affect primarily the nasal cavity, which responds rapidly to triazole antifungal therapy and has a good prognosis. A similar case has only been reported in human medicine in a young adult.

## Background

Histoplasmosis in dogs is usually a systemic disease causing inappetence, weight loss, and fever [1, 2]. Clinical signs of the disease routinely involve the respiratory tract, gastrointestinal tract, and/or other abdominal organs (especially the spleen) [1–3]. Histoplasmosis of the nasal cavity is a rare condition in dogs and cats [4].

*Histoplasma (H.) capsulatum* is a dimorphic fungal organism [2, 5] that is endemic throughout large areas of the temperate and subtropical regions [1, 2] and is commonly found in soil [2, 3]. Infection with *H. capsulatum* is typically via inhalation of microconidia, which are converted to the yeast phase within tissues and then further disseminated through monocytes and macrophages [1, 4, 5]. Long courses of treatment are usually required, and antifungal therapy may be needed for up to 1 year if infection involves tissues with limited drug penetration (bony tissues or the central nervous system). Histoplasmosis is not a contagious disease [2], but direct inoculation or wound infection can transmit the disease from one patient to another. Infection occurs sporadically, recrudescence is possible, and reinfection can occur via inhalation or (less likely) ingestion of the organism from the environment [1, 2]. A varying prognosis is described in dogs with histoplasmosis, with generally better outcomes for those patients with localized (single-organ) rather than systemic *H. capsulatum* infection [1, 2].

The following report describes an apparently immunocompetent dog with nasal histoplasmosis. This case documents the possibility of histoplasmosis in dogs to be localized primarily to the nasal cavity, show a fast response to triazole antifungal therapy, and have a good prognosis. A similar case has only been reported in human medicine in a young adult [6] and nasal involvement was reported in one of 79 dogs in a retrospective case series [7].

## Case presentation

A 4.5-year-old male neutered Cocker spaniel/Poodle mix weighing 7.7 kg (body condition score 6 of 9) was presented with a 3-months history of sneezing, unilateral clear mucoid nasal discharge, and swelling of the left nostril. Previous treatment with antibiotics (clindamycin, enrofloxacin, cefpodoxime, and amoxicillin/ clavulanic acid) and steroids (dexamethasone; 1 single-dose injection [dosage unknown], which 12 days later was followed by 0.03 mg/kg p.o. q48h for 6 days = 3 doses) had not been helpful. The dog also had a right forelimb lameness (grade II-III/IV), the onset of which coincided with that of sneezing and nasal discharge. This had initially responded to analgesia (tramadol) and an over-the-counter joint supplement containing antioxidants but was evidently returned at the time of presentation to the case authors. The dog lived primarily indoors in the Texas Gulf Coast area, and had not travelled outside of this geographic region. A previous diagnosis of an allergic skin disease was managed with a hypoallergenic diet (Royal Canin Duck & Potato). The dog received monthly heartworm prophylaxis and was routinely vaccinated.

**Fig. 1:**
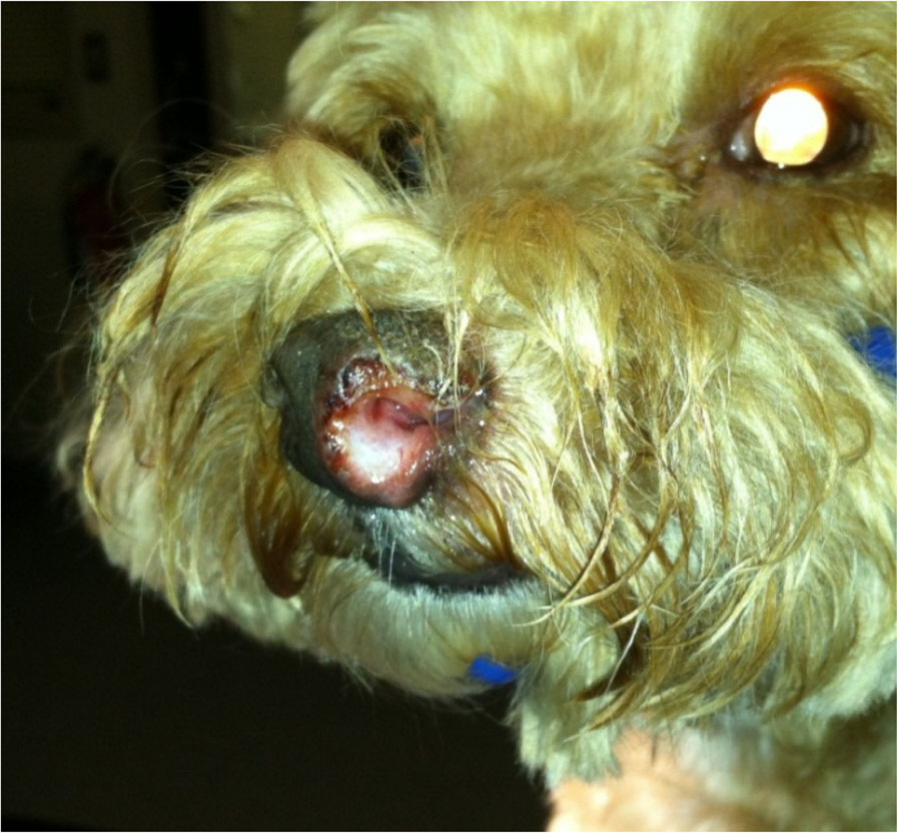
Image of the dog’s nose (predominantly left side) at the initial presentation. The left nostril appeared markedly swollen, ulcerative, deformed, and hypo- to depigmented on physical examination. A serous nasal discharge was noted from both sides. Pain was not evident upon palpation of the nose

**Fig. 2:**
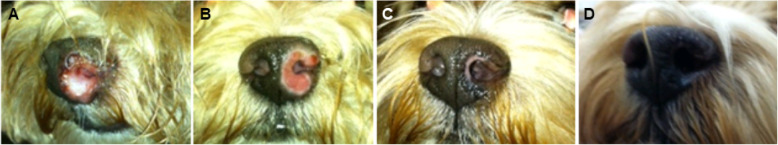
Development of mucocutaneous nasal lesions over a 3-months follow-up period. **a** At the initial presentation, the left nostril was markedly swollen and ulcerative, deformed, hypo- to depigmented, and revealed a clear nasal discharge. A nasal stertor was noted from that side of the nose (**b**) Two weeks (16 days) after initiating treatment with itraconazole, the swelling and ulceration around the left nostril was reduced, with granulation tissue covering the lesion. The dog also no longer had a stertorous breathing. **c** Six weeks later (day 64), the left nostril appeared nearly healed with only a small (approximately 1 mm) rim of depigmentation and granulation tissue around the left nostril. The remainder of the nares was scarred and slightly deformed, but the pigmentation had returned. **d** Four weeks later (day 91), the nasal mucocutaneous lesion had healed with black pigment filling in and around the left nostril, where a one-mm rim of mild scarring was still noticeable

On physical examination, the dog was bright, alert, and responsive but was febrile (rectal body temperature 103.7 °F [39.8 °C], reference interval [RI] for adult dogs: 100.4–102.2 °F [38.0–39.0 °C]). The left nostril was swollen, ulcerative, misshapen, and variably hypopigmented, with a clear nasal discharge (Fig. 1 and 2a). Pain was not evident with palpation over the nasal cavity. Bilateral mild epiphora was present. A mild swelling of the right carpus was also noted, and the dog was resistant to hyperflexion of the right carpal joint (pain score: 2/5). Cardiothoracic auscultation revealed a regularly irregular heart rhythm (respiratory arrhythmia) with a heart rate of 70 beats/minute, a grade II/VI left apical systolic heart murmur, and mildly increased bronchovesicular sounds in all lung fields. Both mandibular lymph nodes were firm and mildly enlarged, symmetrically. All other peripheral lymph nodes were unremarkable. No other abnormalities were noted during the physical examination, which included a rectal examination. Systolic blood pressure, measured by Doppler sphygmomanometry with the patient in right lateral recumbency, was normal (average of three independent measurements: 137 mmHg, RI: 90–140 mmHg).

A complete blood cell count, serum biochemistry profile, urinalysis, and coagulation profile were performed, and revealed a mild lymphopenia (0.51 × 10^9^/L, RI: 1.00–4.80 × 10^9^/L) and thrombocytopenia (manual platelet count: 156 × 10^9^/L, RI: 200–500 × 10^9^/L), mild hyperglobulinemia (40 g/L, RI: 17–38 g/L), moderate hyperfibrinogenemia (6.74 g/L, RI: 1.16–3.64 g/L), and insignificant prolongation of the prothrombin (PT; 7.7 s, RI: 6.0–7.5 s) and partial thromboplastin times (PTT; 10.9 s, RI: 7.1–10.0 s). Buccal mucosal bleeding time was normal (90 s; RI: < 240 s). Urinalysis showed a specific gravity of 1.047, with 30 mg/dL protein (1+ on the urine dipstick) and an inactive urine sediment.

**Fig. 3:**
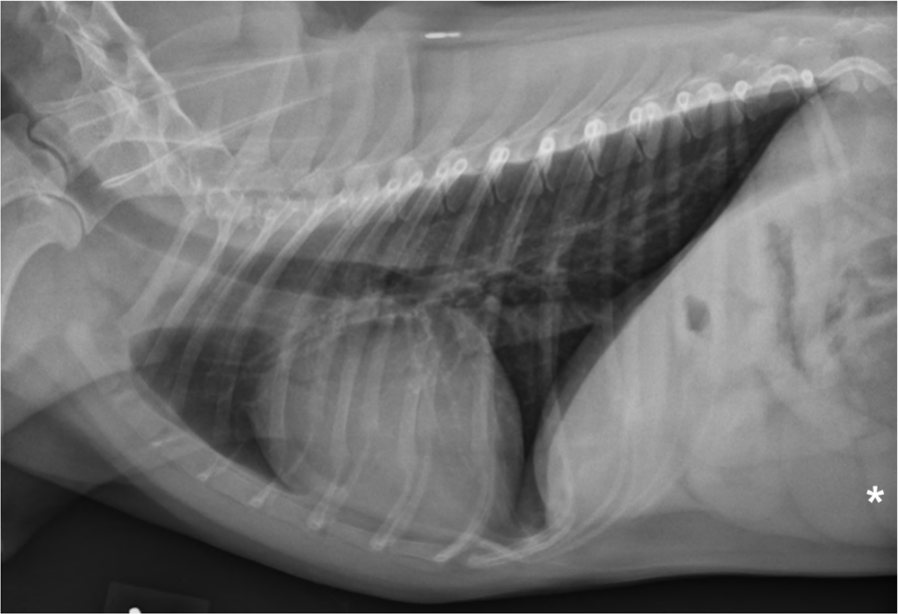
Thoracic radiograph (right lateral view) at the initial presentation. A normal thorax without any evidence of pulmonary infiltrates or lymphadenomegaly is seen. The vertebral heart score (VHS) was normal (10.2, reference: < 10.7). The spleen is visible on the edge of the projection (asterisk) and is enlarged and rounded (splenomegaly)

Diagnostic imaging consisting of thoracic radiographs, computed tomography (CT) scan of the head, and rhinoscopic examination with tissue biopsy was performed to further evaluate the dog for the possibility of a mycotic rhinitis (nasal aspergillosis), immune-mediated disease (Lupus), nasal foreign body, oronasal fistula, or neoplasia, all of which were considered as primary differential diagnoses. Thoracic radiographs (right lateral, left lateral, and ventrodorsal views) were unremarkable, although splenomegaly was noted on the edge of the radiographic projection (Fig. 3). Radiographs of the right carpus indicated an intracapsular swelling from synovitis and joint effusion, with mild erosive changes in the cuboidal bones. Arthrocentesis was not performed.

**Fig. 4:**
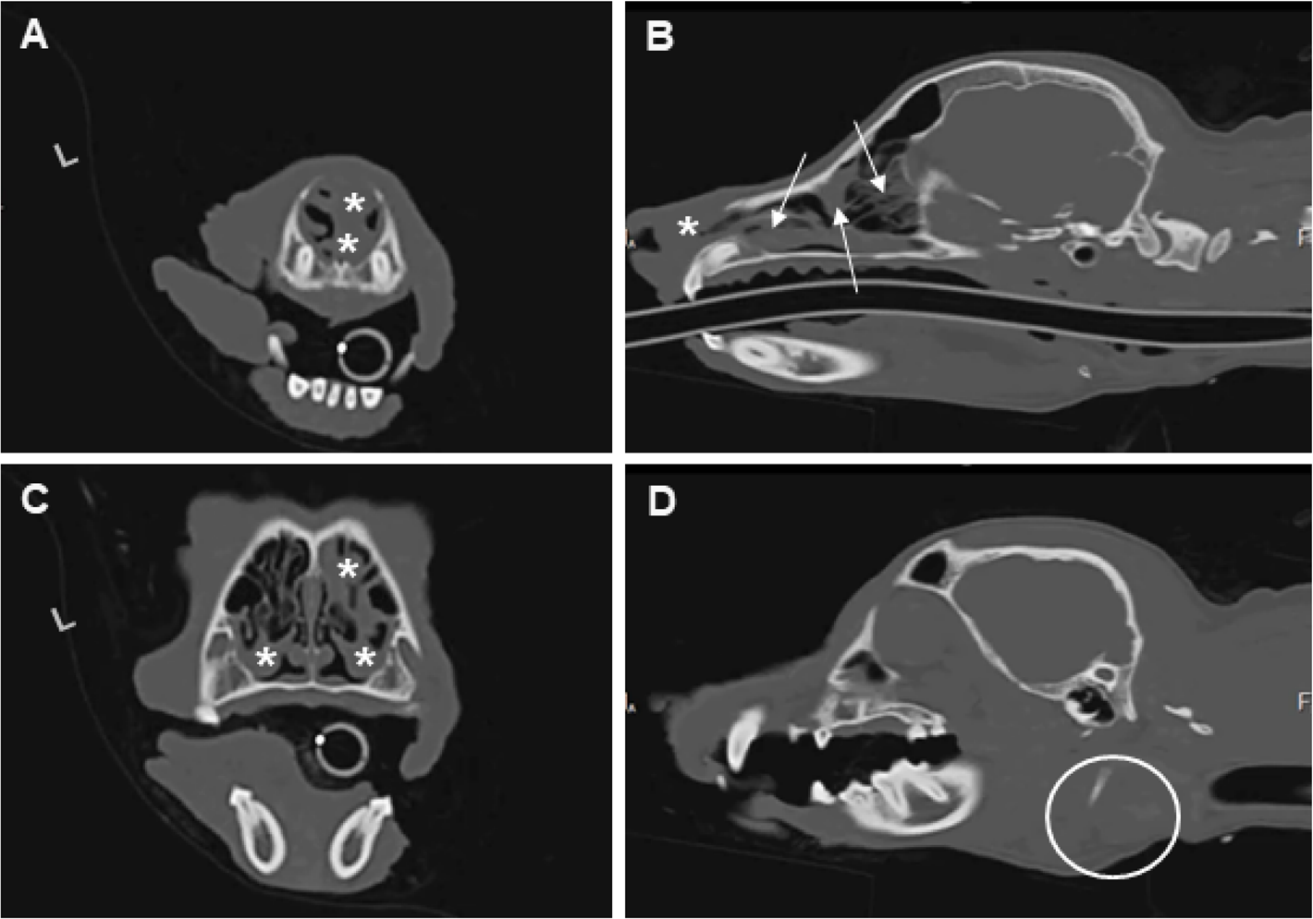
Computed tomography (CT) scan of the head at the initial presentation. Pre-contrast contiguous transverse CT images (dorsal plane: **a** and **b**; and sagittal plane: **c** and **d**) revealed soft tissue-attenuating material in both rostral nares (**a**, **c**; asterisks), which appeared obstructed rostral to the maxillary incisor teeth (**b**; asterisk), thickened soft tissues of the left nasal planum, and a collection of soft tissue-attenuating material within the nasal cavity caudal to the maxillary incisors and intermixed with the nasal turbinates (**b**; arrows). Mandibular and medial retropharyngeal lymph nodes were enlarged (**d**; circle), but there was no evidence of bone lysis. No abnormalities located to the frontal sinuses were detected

**Fig. 5:**
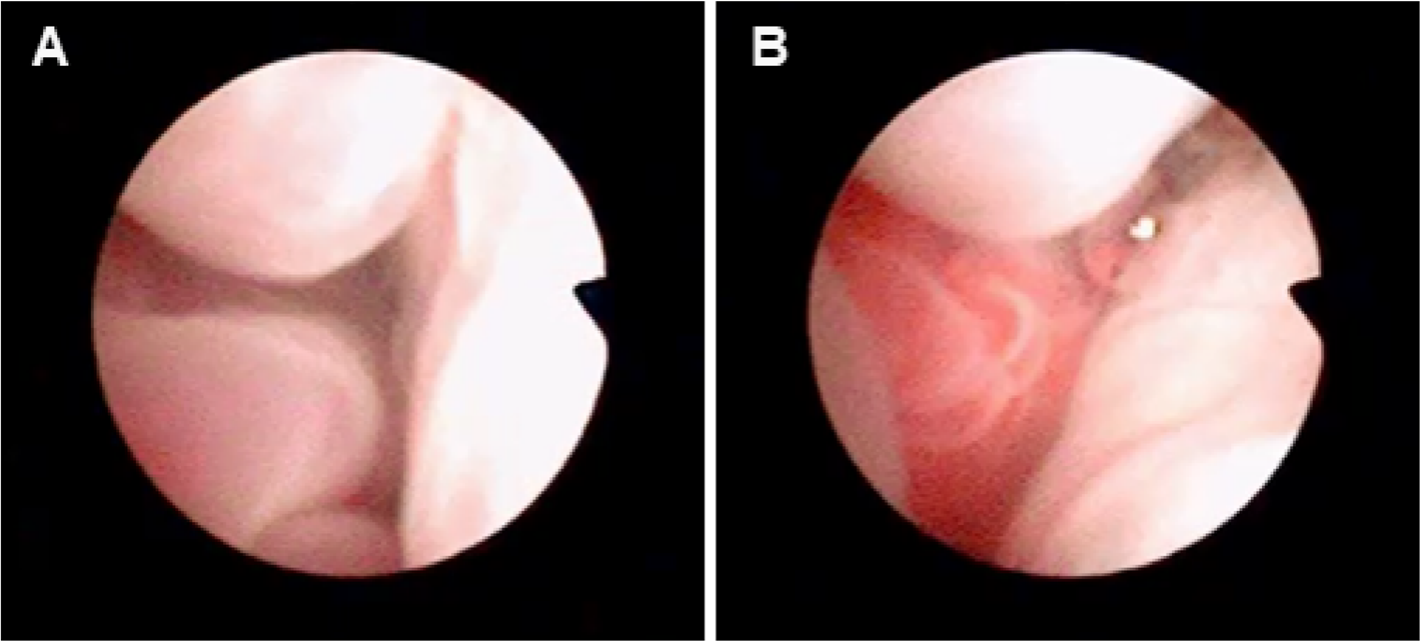
Rhinoscopy of the nasal passages. Mucosal swelling (**a**) and tissue proliferation (**b**) were seen upon rhinoscopic evaluation of both nasal cavities. Endoscopic tissue biopsies were obtained from the medial and lateral portions of both nasal cavities

A pre- and post-contrast computed tomography (CT) scan of the head showed evidence of swelling and increased tissue density localized to the rostral portion (approximately 1.5 cm) of both the left and right nasal passages, with bilateral mandibular and medial retropharyngeal lymphadenopathy (Fig. 4). These findings were consistent with a diagnosis of a neoplastic (squamous cell carcinoma, melanoma, or other neoplasia), infectious (e.g., mycosis), or inflammatory disease process (e.g., chronic idiopathic rhinitis). Rigid rhinoscopy was used to evaluate further the left and right nares and nasal passages (Fig. 5). Blind tissue biopsies (*n* = 11) were obtained from the medial and lateral aspects of both nares.

**Fig. 6:**
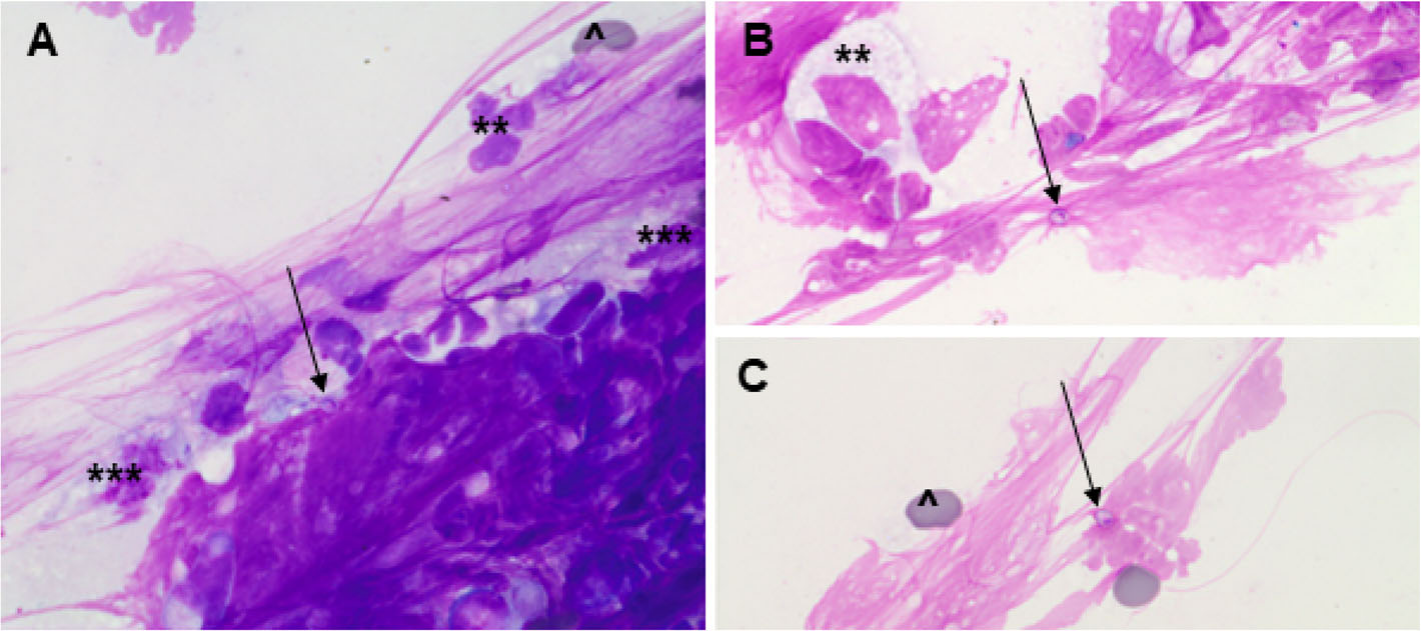
Cytologic examination of an impression smear from a biopsy obtained from the left nares. The smears are of low cellularity with (**a**, **b**) few nucleated cells: variably degenerate neutrophils (**) and macrophages (***), with epithelial cells and occasional small lymphocytes (not shown) and (**c**) rare erythrocytes (^) in a lightly proteinaceous background with streaming mucoid material. Pale blue structures (approximately 2 × 4 μm) with a peripheral pink nuclear-type structure and a thin, clear capsule – consistent with *Histoplasma capsulatum* (yeast form) – are rarely seen extracellularly (arrows)

**Fig. 7:**
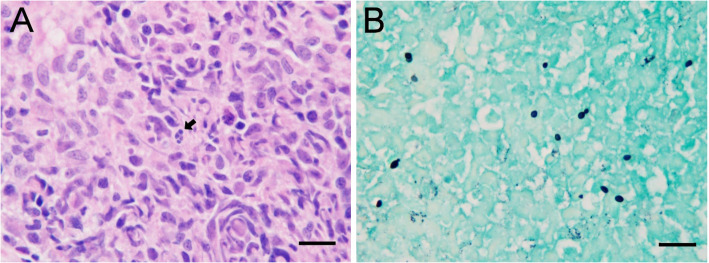
Histologic examination of a biopsy of the left nares. The nasal submucosa was expanded by numerous epithelioid macrophages that rarely contained individual and clusters of 1–2 μm, round yeasts (panel **a**; arrow). Narrow-based budding was observed on GMS stain (panel **b**). **a** Hematoxylin and eosin stain, 100x oil objective. **b** Gömöri methenamine (GMS) silver stain, 100x oil objective; bar = 5 μm

Cytology of an impression smear made from a biopsy showed evidence of neutrophilic and histiocytic inflammation with the presence of extracellular yeast organisms consistent with *Histoplasma capsulatum* (Fig. 6). Histologic examination revealed a moderate to severe diffuse, histiocytic, and chronic suppurative rhinitis with intralesional yeast organisms (Fig. 7).

Further diagnostic evaluation of the dog for evidence of systemic histoplasmosis included abdominal ultrasonography, fundic examination, and the *H. capsulatum* antigen test. Abdominal ultrasonography revealed a mild hepatic hypoechogenicity and mild splenic enlargement. Fine-needle aspirates were not performed. Fundic examination was unremarkable. A quantitative sandwich enzyme-linked immunosorbent assay (ELISA) for *H. capsulatum* antigen (MiraVista Diagnostics, Indianapolis, IN, USA) [8] was performed using both serum and urine samples; this was negative with serum but weakly positive with urine (0.58 ng/mL).

Treatment was initiated on the day after presentation and diagnostic evaluation (day 1), with itraconazole at 13 mg/kg PO q24h (Itraconazole®; Patriot Pharmaceuticals, Horsham, PA, USA) given on an empty stomach [9]. In addition, tramadol 3.3 mg/kg PO q8h was administered for 3 days following the diagnostic work-up.

Two weeks later (day 17), the dog showed significant clinical improvement. The nasal discharge had resolved, and the left nostril was in the process of healing, with granulation tissue circumferentially present (Fig. 2b). The swelling of the right carpus had also resolved. At this time, the *H. capsulatum* antigen test (MiraVista) was negative with both serum and urine (< 0.1 ng/mL). The dose of itraconazole was decreased to 8 mg/kg PO q24h because the serum biochemistry profile revealed a moderate increase in serum alanine aminotransferase (ALT) activity (366 U/L, RI: 10–130 U/L). With the suspicion of itraconazole-induced (intrinsic) hepatotoxicity, S-adenosylmethionine/silybin (Denamarin®, Nutramax, Edgewood, MD, USA) was added to the dog’s treatment plan (225 mg/24 mg PO q24h). Serum ALT activity was rechecked 3 weeks later (day 38), at which point it had normalized (110 U/L).

A follow-up evaluation 6 weeks later (day 64) showed further clinical improvement. The left nostril appeared nearly healed (Fig. 2C), peripheral lymph nodes were normal in size and shape, and the dog was no longer lame. The *H. capsulatum* antigen test (MiraVista) was again negative with serum and urine, and the serum ALT activity remained within the reference range (79 U/L). Itraconazole (8 mg/kg PO q24h) and S-adenosylmethionine/silybin (Denamarin®) were continued.

At another follow-up evaluation 4 weeks later (day 91), nasal mucocutaneous histoplasmosis was considered as resolved (Fig. 2d). The *H. capsulatum* antigen test (MiraVista) was negative with serum and urine (< 0.1 ng/mL), and the serum ALT activity was 124 U/L. Radiographs were performed on the on the right and left carpi. The soft tissue swelling surrounding the right carpal region had resolved. However, the radiolucent areas of the distal medial aspect of the right intermedioradiocarpal bone and the right fourth carpal bone were unchanged. As the appearance of these bones on the left (clinically unaffected) side was very similar, these findings were considered to be normal anatomic variants rather than lytic lesions. Thus, itraconazole (8 mg/kg PO q24h) and S-adenosylmethionine/silybin (Denamarin®) were given for another 4 weeks and then discontinued.

The dog was known to be alive with no apparent evidence of recrudescence of infection more than 6 years after this last examination.

## Discussion and conclusions

We report a case of subacute to chronic mucocutaneous histoplasmosis in a dog without evidence of underlying immunocompromise. Geographically, this dog lived in the Texas Gulf Coast area, a temperate region where the thermally dimorphic fungus *H. capsulatum* is endemic and is commonly found in the environment [10, 11]. Potential sources of infection in this case include household dust or contaminated soil (e.g., potted plants, flower beds) [3]. An increased risk of infection has also been reported with the renovation of older buildings or basements [1, 3, 12, 13], which was not obvious from the patient history. The true prevalence of histoplasmosis in companion animals in this geographic region is difficult to determine because of the endemic status of the fungal organism [2, 7, 14].

An increased risk for severe clinical disease following *H. capsulatum* infection is reported in immunosuppressed individuals [1–3], although – in contrast to human patients – many dogs with clinical signs related to infection with *H. capsulatum* are not known to be immunosuppressed [7]. Infection of dogs typically occurs via inhalation or – less commonly – via ingestion of *H. capsulatum* spores [1–3, 15, 16].

Histoplasmosis has been reported in dogs ranging in age from 2 months to 14 years, but most affected dogs are younger than 5 years of age [2, 7, 17]. At 4.5 years of age, the dog in this case was in the age range – but not the typical breed or breed type – of dogs with an increased risk for contracting systemic histoplasmosis [7, 17]. The dog presented at the end of July with a three-month history of clinical signs. Thus, the infection likely happened in the spring, which concurs with the seasonal spike of histoplasmosis cases in dogs [17].

The case presented shows that cytological and/or histological examination of tissue specimens are suitable for diagnosing nasal histoplasmosis. Cytological examination of the affected tissues in this case suggested infection with *H. capsulatum*, although the yeast forms were only noted extracellularly. True infection, rather than contamination with environmental *H. capsulatum* organisms, was subsequently confirmed by both histopathological examination – where yeast forms of *H. capsulatum* were present within macrophages – and antigen detection via ELISA [15, 18].

Routine hematology and serum biochemistry profiles are typically normal with mycoses affecting the nasal cavity [4]. Peripheral eosinophilia may be seen with fungal infections (particularly aspergillosis) but was not present in this case. Thrombocytopenia and hyperglobulinemia, as seen in this case, are common in dogs with histoplasmosis [1, 2, 7].

The MVista *H. capsulatum* quantitative antigen ELISA (MiraVista) has been validated for use in dogs and can be performed on serum or urine. This quantitative test detects a component of *H. capsulatum* galactomannan, a component of the fungal cell wall. The test has a high sensitivity (95%) in dogs [8, 16], but false-negative results are possible, particularly with localized histoplasmosis [12, 16]. A higher sensitivity has been reported for the urine antigen test compared to the detection of antigenemia in people [8, 19], dogs [16], and cats [15], and this finding is consistent with the results reported for the patient described here. In human patients, lower antigen levels are routinely noted in immunocompetent versus immunocompromised individuals, those with a localized versus disseminated disease, and patients with subacute or chronic infection versus acute histoplasmosis [19].

Cross-reactivity of the MVista *H. capsulatum* antigen test with other endemic mycoses is reported [19]. Although possible, cross-reactivity with *Blastomyces dermatitidis* is very unlikely in this case given both the cytological findings and the geographical location. *H. capsulatum* produces a small (2–4 μm diameter) yeast with a thin and clear capsule, whereas the yeast form of *B. dermatitidis* is larger (8–15 μm diameter) and can demonstrate broad-based budding [1, 20]. Also, blastomycosis is not prevalent in the Texas Gulf Coast area [20]. PCR analysis would have been most specific for fungal identification [1, 15], but it is not routinely available. Fungal culture (which takes over 10–20 days) was not performed due to the high zoonotic risk. Still, potential cross-reactivity of the antigen test is of minor clinical relevance as similar antifungal treatment regimens are used for both infections [1–4, 20, 21].

Localized bone lysis is typically seen in dogs with sino-nasal aspergillosis, whereas intact nasal turbinates were seen on CT in the dog described in this report. Lameness, arthralgia, and joint swelling have been reported in dogs with *H. capsulatum* infection [1, 16]. Whether this is due to the presence of *H. capsulatum* organisms within musculoskeletal structures or reflects a secondary immune-mediated response is unclear, although a series of cats with bone and joint infection was recently reported [22]. The rapid resolution of lameness and swelling with antifungal treatment in the dogs reported here would be compatible with either mechanism, but the recurrent nature of the clinical signs would favor a secondary immune-mediated etiology.

A varying prognosis in dogs with histoplasmosis is described [1]. Survival rates for dogs with pulmonary, gastrointestinal, or disseminated histoplasmosis range from 33 to 78%; better outcomes are generally reported for patients with localized disease [2, 7]. The rapid and sustained response to treatment in the dog described here suggests that nasal mucocutaneous infection may be highly amenable to treatment and conveys a good prognosis.

The treatment of choice for histoplasmosis is systemic or topical triazole antifungal therapy (itraconazole or fluconazole) and/or amphotericin B, and long courses of antifungal treatment (at least 4–6 months) are usually required [1, 2, 4, 7, 9, 13, 21, 23]. Itraconazole is widely regarded as the preferred choice, and appears to be a safe and efficacious treatment option for nasal histoplasmosis [9, 23]. This triazole blocks fungal ergosterol synthesis leading to increased cell membrane permeability and interference with intracellular processes [9]. The long half-life of itraconazole (51 h) allows for once-daily dosing with higher steady-state concentrations [9] but an increased risk of adverse effects. Oral bioavailability of itraconazole capsules is approximately 20% in dogs [9], and generic pelletized itraconazole capsules as used in this case are pharmacokinetically similar to brand-name itraconazole capsules [21, 24]. In the case presented here, this treatment resulted in a significant clinical improvement within 13 weeks (3 months). However, the effects of treatment were likely much longer given the high volume of distribution and long half-life of itraconazole [9].

Hepatic injury appears to be a dose- and duration-dependent side effect of itraconazole treatment [1, 3, 9]. Periodic evaluation of serum liver enzyme activities (especially hepatic leakage enzyme activities) and liver substrates is therefore recommended during itraconazole treatment [3, 7, 9, 25]. In the case presented, serum ALT activity increased to 2.8-times above the upper reference limit 2 weeks after starting itraconazole treatment but normalized in 3 weeks following a 40% dose reduction (13 mg/kg q24h to 8 mg/kg q24h). The lower dose was achieved by resizing the pellet-containing original capsules by weighing to obtain lower-dose capsules [21]. This approach was preferred over switching to the brand-name itraconazole liquid solution due to the costs involved. Therapeutic drug monitoring [21] was not performed in this patient, thus information regarding trough levels achieved with the lower dose was not available.

Ulceration of the external nares is routinely noted in dogs with fungal infection within the nasal cavity [4, 26]. Nasal infection with *H. capsulatum* is unusual in dogs and cats [1, 4, 7, 16], and involvement of the mucocutaneous area has not been described. This patient’s history and disease course led us to a presumptive diagnosis of primary nasal histoplasmosis. However, the possibility of an underlying disease (e.g., idiopathic lymphoplasmacytic rhinitis) or an injury to the nose cannot be discounted. It is also possible that prior antimicrobial and/or anti-inflammatory treatment predisposed this dog to subsequent infection with *H. capsulatum*. As previously reported, this case supports that antibiotic treatment should be used sparingly in nasal discharge cases in dogs [26].

Histoplasmosis typically presents as a systemic mycosis in dogs [1, 2, 7]. While the disease appeared to be localized primarily to the nasal cavity in the case presented, carpal swelling and lameness, as well as mild splenomegaly might have indicated extra-nasal involvement. Absent the results of any further diagnostics (e.g., synovial and/or splenic aspirates), this possibility can neither be confirmed nor excluded. This dog’s mild initial antigenuria (< 4 ng/mL [19]), along with his rapid and sustained clinical resolution with only 3 months of antifungal treatment, make a disseminated mycosis less likely, as systemic infections typically require longer courses of treatment [1–3].

Monitoring of *H. capsulatum* antigen levels has been reported as effective means to monitor response to treatment in human and veterinary patients with histoplasmosis [8, 27]. It is noteworthy that this dog’s *H. capsulatum* urine antigen test was negative after just 2 weeks of itraconazole. The authors attributed this to the localized nature of the infection, and therefore relied on the appearance of the affected area to guide treatment decisions.

Because the test was repeatedly negative during follow-up evaluations, the improvement and resolution of clinical signs appeared to be the best means of monitoring the dog during treatment with itraconazole.

We acknowledge a few limitations to this case report. Most importantly, diagnostics pertaining to the concurrent lameness were limited to radiographic studies. Infection of the carpal bones or joints with *H. capsulatum* cannot therefore be excluded. Carpal arthrocentesis with joint fluid cytology may have provided additional useful information, as organisms were routinely identified in synovial fluid in a recent series of cats with histoplasmosis and bone or joint involvement [22]. In addition, it is the opinion of the authors that this dog was not immunocompromised, as he had not received any immunomodulatory therapies prior to the onset of clinical signs and appeared otherwise systemically well. However, an occult disease process impacting immune responses (e.g., a congenital immunodeficiency syndrome such as selective immunoglobulin deficiency) cannot be completely excluded. During the dog’s physical examination, a heart murmur (grade II/VI) was noted and presumed to reflect a mitral valve regurgitant jet due to degenerative mitral valve disease or mitral valve dysplasia. Further diagnostics (i.e., echocardiogram, systemic blood pressure) to evaluate the murmur were recommended but declined by the owner. Concurrent endocarditis was therefore not excluded, but seems highly unlikely.

This case suggests that dogs with nasal mucocutaneous histoplasmosis may respond well to systemic antifungal therapy, and experience excellent long-term outcomes. *H. capsulatum* antigenemia and antigenuria may be absent or modest, and clinicians must therefore rely on the appearance of the affected area to guide treatment decisions.

## Data Availability

Data sharing does not apply to this article as no datasets were generated or analyzed during the current study. Further data and information about this case is available from the corresponding author upon reasonable request.

## Abbreviations

ALT: Alanine aminotransferase
CT: Computed Tomography
PO: Per Os
